# Assessment of oral self-care in patients with periodontitis: a pilot study in a dental school clinic in Japan

**DOI:** 10.1186/1472-6831-9-27

**Published:** 2009-10-29

**Authors:** Atsushi Saito, Momomi Kikuchi, Fumie Ueshima, Shinya Matsumoto, Hiroki Hayakawa, Hitomi Masuda, Takemi Makiishi

**Affiliations:** 1 Department of Clinical Oral Health Science, Tokyo Dental College, 2-9-18 Misaki-cho, Chiyoda-ku, Tokyo, 101-0061 Japan; 2 Section of Dental Hygiene, Suidobashi Hospital, Tokyo Dental College, 2-9-18 Misaki-cho, Chiyoda-ku, Tokyo, 101-0061 Japan

## Abstract

**Background:**

Oral hygiene education is central to every stage of periodontal treatment. Successful management of periodontal disease depends on the patient's capacity for oral self-care. In the present study, the oral self-care and perceptions of patients attending a dental school clinic in Japan were assessed using a short questionnaire referring to existing oral health models.

**Methods:**

A cross-sectional study design was used. The study population consisted of sixty-five patients (age range 23-77) with chronic periodontitis. The pre-tested 19-item questionnaire comprised 3 domains; 1) oral hygiene, 2) dietary habits and 3) perception of oral condition. The questionnaire was used as a part of the comprehensive assessment.

**Results:**

Analyses of the assessment data revealed no major problems with the respondents' perceived oral hygiene habits, although their actual plaque control levels were not entirely adequate. Most of the respondents acknowledged the importance of prevention of dental caries and periodontal diseases, but less than one third of them were regular users of the dental care system. Twenty-five percent of the respondents were considered to be reluctant to change their daily routines, and 29% had doubts about the impact of their own actions on oral health. Analyzing the relationships between patient responses and oral hygiene status, factors like 'frequency of tooth brushing', 'approximal cleaning', 'dental check-up' and 'compliance with self-care advice' showed statistically significant associations (*P *< 0.05) with the plaque scores.

**Conclusion:**

The clinical utilization of the present questionnaire facilitates the inclusion of multiple aspects of patient information, before initiation of periodontal treatment. The significant associations that were found between some of the self-care behaviors and oral hygiene levels document the important role of patient-centered oral health assessment in periodontal care.

## Background

Periodontal disease, a major oral health problem, reportedly affects more than 80% of the adult population in Japan [1]. When left untreated, periodontitis often causes tooth loss that can place significant burden on individuals [2]. Furthermore, periodontitis has been implicated as an emerging risk factor for a number of major systemic diseases or conditions, including cardiovascular disease, stroke, and diabetes, as well as for pre-term, low-birth weight infants [3-5]. Prevention of and early intervention into periodontal disease is critical, and oral hygiene education is central to all stages of treatment. In addition to professional care, successful management of periodontal disease depends on the capacity of patient's oral self-care [6,7]. A change in patient attitude and behavior is often desirable when periodontitis is treated. The information gathered relative to a patient's values and beliefs may be a useful guide in designing effective oral health care interventions [8].

Research into modern approaches to health education on motivating individuals or groups has been done. Conceptual models such as Health Belief Model (HBM) [9], Self- efficacy [10-12], and Health Locus of Control (LOC) [13,14] have shown some promise. Instruments based on these models have been clinically examined for their abilities to explain oral health habits, oral hygiene and periodontal parameters.

In order to plan effective interventions which encourage patient self-care, it is first important to collect basic information regarding self-care behavior and perception. Currently, there is no universally accepted or recommended assessment tool for oral health behavior of periodontitis patients. Because of the limited amount of time available at clinical appointments, utilizing complex instruments based on health behavior models may not always be practical. Thus, there is a need for concise assessment of such data. In this study, the Client Self-care Commitment model (CSCCM) [15] and the New Century model of oral health promotion [16] were used as theoretical frameworks for the assessment. The aim of this exploratory study was to assess oral self-care and perceptions of patients with periodontitis, using a short questionnaire incorporating the essence of the oral health conceptual models.

## Methods

### Subjects

A cross-sectional study design was used. Subjects were recruited from patients who visited the Suidobashi Hospital, Tokyo Dental College, Tokyo for initial assessment and treatment of periodontitis. Ethics approval was granted by the institutional ethics committee, and the patients gave written informed consent to participate in this study.

Subjects were invited to participate if they were clinically diagnosed with mild to moderate chronic periodontitis. Inclusion/exclusion criteria consisted of the presence of a minimum of four ≥ 4 mm probing depth in different quadrants with radiographic evidence of bone loss, the presence of ≥ 20 teeth with a minimum of 4 molars, no extensive periodontal therapy in the previous 6 months, and good general health (as assessed by the recruiting clinician).

### Data collection

Before commencement of initial periodontal therapy, data regarding behavior and perception of self-care were collected by means of clinical oral examination and a self administered questionnaire.

#### Clinical oral examination

After collection of full medical and dental histories, a periodontal examination was carried out. A total of 6 clinicians (4 dentists and 2 dental hygienists) performed the initial examination. Before the clinicians took part in the study, they were given detailed instructions and underwent comparative examinations with result assessments. The following periodontal parameters were recorded at six sites for each tooth. Probing depth (PD) was measured using a Williams probe with a force of 0.3 N by the examiner rounding up to the nearest millimeter. Full-mouth bleeding scores [17] were recorded as the presence or absence of bleeding following measurement of PD. Several tooth-sites were excluded from the examination; impacted teeth, retained roots, grossly broken down teeth, teeth which were too inaccessible to examine satisfactorily. The presence or absence of supragingival dental plaque was recorded by Plaque Control Record (PCR) of O'Leary et al. [18].

#### Questionnaire

The domains and items of the questionnaire were generated through literature review and the use of conceptual models. From the review of the literature [6,19,20], several behaviors were identified as key to oral hygiene and self-care. Beliefs and attitudes associated with these oral health behaviors were developed based mainly on the conceptual models of CSCCM [15] and the New Century model of oral health promotion [16], and presented in a questionnaire format (3 printed pages). The base instrument was piloted with 67 volunteers during the simulated patient practice at a dental hygiene program for content validity [21]. A peer-focus group of one periodontist and two dental hygienists with extensive clinical experiences further refined the instrument. The final, refined version is composed of 3 domains; 1) oral hygiene, 2) dietary habits and 3) perception of oral condition, with a total of 19-item questions (Table 1). Each question is provided with various levels of agreement or categorical options for the patient to choose from. The clinicians supervised the patients during the completion of the questionnaires to ensure that all questions were understood.

**Table 1:** The domains and item questions of the questionnaire (translated in English)

**Domains**	**Items**
Oral hygiene	1. Frequency of toothbrushing
	2. Use of toothpaste
	3. Approximal cleaning
	4. Use of mouthwash or other products

Dietary habit	1. Frequency of meal
	2. Self evaluation of diet
	3. Frequency of between-meal snacks
	4. Types of snacks

Perception of oral condition	1. How often do you check your teeth or mouth inside in a mirror?
	2. How would you rate your desire to keep your teeth?
	3. What are your hopes for your oral health?
	4. How much are you willing to do to improve your oral health?
	5. What actions you are likely to take to improve your oral health?
	6. Have you been maintaining regular dental check-ups?
	7. How much do you follow your dentist's or dental hygienist's advice on oral hygiene care?
	8. How important is the prevention of cavities or gum diseases for you?
	9. Would you like your dentist or dental hygienist to recommend oral care products?
	10. Are you willing to take new challenges and/or change your daily routine?
	11. How do you perceive consequences of your actions on oral health?

### Data Management and Statistical Analyses

In preparation for analyses, data were entered into an Excel spreadsheet and proofed for data-entry errors. The responses to questionnaire items were dichotomized based mainly on the frequency of responses, since we had no prior knowledge on a cut-off reflecting qualitative differences. The Fisher's exact test was used to assess the relationship between frequencies of the response variables and oral hygiene status. For all statistical calculations, the InStat 3.0 software package (GraphPad, La Jolla, CA) was used. All reported *P*-values are two-tailed, and *P*-values less than 0.05 were considered statistically significant.

## Results

A total of 65 patients (23 males and 42 females; 23 to 77 years of age; mean age: 54 ± 14 years) agreed to participate and responded to the questionnaire. Approximate time required for a participant to fill out the questionnaire ranged from 5 to 13 min. Clinical and demographic characteristics of the subjects are shown in Table 2. The participants had an average of 26 remaining teeth.

**Table 2:** Demographic characteristics and clinical parameters of subjects at baseline (total n = 65)

Gender	
Male (n)	23
Female (n)	42
Age^†^	54 ± 14
Age distribution	23-77
No. of teeth^†^	26 ± 3
PD^†^	3.48 ± 0.91
% sites with bleeding^†^	26.8 ± 22.5
% sites with PD ≥ 4 mm^†^	31.8 ± 23.2
PCR (%)^†^	49.5 ± 21.6

^†^Mean ± SD

### Oral hygiene

Under the domain of oral hygiene, the questionnaire asked about frequency of tooth brushing, use of dentifrice, approximal cleaning, and use of mouthrinse and other adjunct products (Table 3). Altogether, sixty percent of respondents indicated brushing three times more a day, while 6% indicated brushing only once a day. A majority of respondents were using dentifrice. Two thirds of respondents indicated that they clean proximal surfaces regularly, with interdental brush or dental floss. Those using mouthrinse or other adjunct oral care products were 38%.

**Table 3:** Summary of responses to "Oral hygiene" and their relationship with plaque score

**Oral hygiene Items**	**Responses and binarization**†	**Relationship with plaque score**
1. Frequency of brushing (per day)		*P *= 0.0354
none	0 (0)	
1x	4 (6)	
2x	22 (34)	
		
3x	29 (45)	
4x or more	10 (15)	

2. Use of tooth paste		ND
Yes	61 (95)	
No	3 (5)	

3. Approximal cleaning (use of dental floss or interdental brush)		*P *= 0.0232
Yes	39 (60)	
		
No	26 (40)	

4. Use of mouthwash or other products		NS
Yes	25 (38)	
		
No	40 (62)	

Values are expressed in n (%).

† Responses were grouped into two (as separated by dotted line) and compared with two categories of PCR (≥ 40% vs. < 40%) by Fisher's exact test. *P*-values less than 0.05 were considered statistically significant.

NS; not significant

ND; not determined

In order to analyze the relationship with plaque scores, the individual PCR data was dichotomized. The cut-off value of the PCR for dichotomization was set at 40%. We decided that this value was appropriate considering (1) mean value of the PCR score, and (2) that the participants were at the pre-treatment stage. When the relationship with plaque score was sought, frequency of brushing and approximal cleaning showed a statistically significant association (*P *< 0.05) (Table 3). The habit of brushing three times more a day was associated with better PCR scores. Likewise, the habit of approximal cleaning was associated with better PCR scores.

### Dietary habits

In general, no apparent problems with dietary habits were identified in this population of patients. As for frequency of meals, a majority of respondents (86%) reported eating three meals a day with the rest eating two meals a day (Table 4). Over two thirds of respondents reported snacking between meals at least once a day. As for respondents' self evaluation of their dietary status, 87% rated their own habits as good or fair, while 13% rated them as poor.

**Table 4:** Summary of responses to "Dietary habits" and their relationship with plaque score

**Dietary habits Items**	**Responses and Binarization**†	**Relationship with plaque score**
1. Frequency of meals (per day) n = 65		NS
none	0 (0)	
1x	0 (0)	
2x	9 (14)	
		
3x	56 (86)	
4x or more	0 (0)	

2. Self-evaluation of diet n = 61		NS
Good	30 (49)	
Fair	23 (38)	
		
Bad	8 (13)	

3. Frequency of between-meal snacks (sweets) n = 55		NS
none	14 (25)	
		
1x	27 (49)	
2x	13 (24)	
3x	1 (2)	
4x or more	0 (0)	

Values are expressed in n (%). Items subjected to the statistical analysis were shown.

†Responses were grouped into two (as separated by dotted line) and compared with two categories of PCR (≥ 40% vs. < 40%) by Fisher's exact test. *P*-values less than 0.05 were considered statistically significant.

NS; not significant

None of the items showed a significant association with plaque scores (Table 4.)

### Perception of oral condition

A summary of the responses is shown in Tables 5 and 6. When asked to indicate the frequency of oral self-examination (looking into one's own mouth), 66% of respondents reported doing so at least once a week (Table 5). A majority (74%) of respondents indicated a strong desire to keep their teeth for as long as possible. When asked to report other things they desired in relation to oral health, 'eating enjoyment' and 'fresh breath' were the most frequent answers. Two thirds of respondents indicated that they would do anything to improve their oral conditions. 'Implementation of the suggested oral care regimen' and 'acceptance of necessary dental care' were the most frequently reported behaviors or attitudes that they would be likely to accept. Less than one third of respondents were regular users of the dental care system (i.e., they had at least one dental visit within 1 year).

**Table 5:** Summary of responses to item 1 to 6 of "Perception of oral condition" and their relationship with plaque score

**Perception of oral condition**	**Responses and binarization**†	**Relationship with plaque score**
**Items**		
1. How often do you check your teeth or mouth in a mirror? n = 65		NS
everyday	22 (34)	
a few times/week	21 (32)	
		
a few times/month	11(17)	
almost never	11 (17)	

2. How would you rate your desire to keep your teeth? n = 65		NS
Very strong	48 (74)	
		
Strong	15 (23)	
Fair	2 (3)	
Weak	0 (0)	
Very weak	0 (0)	

4. How much are you willing to do to improve your oral health? n = 65		NS
Anything necessary	40 (62)	
		
I am willing to take some action.	25 (38)	
Not very much	0 (0)	

6. Have you been maintaining regular dental check-ups? n = 63		*P *= 0.0407
Yes	17 (27)	
		
No	46 (73)	

Values are expressed in n (%). Items subjected to the statistical analysis were shown.

†Responses were grouped into two (as separated by dotted line) and compared with two categories of PCR (≥ 40% vs. < 40%) by Fisher's exact test. *P*-values less than 0.05 were considered statistically significant.

NS; not significant

**Table 6:** Summary of responses to item 7 to 11 of "Perception of oral condition" and their relationship with plaque score.

**Perception of oral condition Items**	**Responses and binarization**†	**Relationship with plaque score**
7. How much do you follow your dentist's or dental hygienist's advice on oral hygiene care? n = 65		*P *= 0.0101
Always	21 (33)	
Sometimes	27 (42)	
		
Not very much	5 (8)	
Never	11 (17)	

8. How important is the prevention of cavities or gum diseases for you? n = 65		NS
Very important	58 (89)	
		
Somewhat important	7 (11)	
Not at all important	0 (0)	

10. Are you willing to take on new challenges and/or change your daily routine? n = 63		NS
Yes	47 (75)	
		
No	16 (25)	

11. To what degree do you feel that the actions you take have an impact on your own oral health? n = 63		NS
My actions play a significant role	45 (72)	
		
Not sure either way	16 (25)	
I cannot do much about it	2 (3)	

Values are expressed in n (%). Items subjected to the statistical analysis were shown.

† Responses were grouped as shown and compared with two categories of PCR (≥ 40% vs. < 40%) by Fisher's exact test. *P*-values less than 0.05 were considered statistically significant.

NS; not significant

Seventeen percent of the respondents indicated that they never followed or received professional advice on oral care, while everyone acknowledged the importance of prevention (Table 6). A majority of them indicated that they would like to receive professional advice on oral care products.

A quarter of respondents did not wish to take on new challenges or change their daily routines. Seventy one percent of them believed that their actions would influence their oral health, while 3% felt they could not.

When the relationship with plaque score was sought, maintenance of regular dental check-ups and acceptance of professional advice were the items which showed a significant association with PCR scores. Those who had regular dental check-ups had better PCR scores. Also, those who accepted dentist's or dental hygienist's advice on oral hygiene care had better PCR scores.

## Discussion

In an age of assessment and accountability, the field of periodontics as well as dental hygiene could benefit by adopting models that emphasize the multidimensional nature of oral problems and by considering patient behaviors and perceptions on oral health.

Although it may be desirable to use a full instrument based on single or multiple health behavior models for a comprehensive assessment, clinicians often experience time constraints posed by active patient care. Therefore, we sought to develop a simple questionnaire that could be completed quickly, yet it would provide information relevant to the planning of periodontal treatment. Our 19-item questionnaire imposed very little burden on the patients; they had little difficulty in completing it. Within the limitations of the present study, the clinical use of the questionnaire disclosed salient information regarding periodontitis patients' oral health behavior and perceptions.

To prevent development of dental caries and gingivitis the usual recommendation is to brush the teeth twice a day and clean interdentally at least once a day [22,23]. In a recent national survey of the Japanese population, brushing more than twice-a-day was reported by 70% of those surveyed [1]. In the present study, all participants reported that they brush their teeth at least twice a day, and 60% indicated that they clean interdentally. However, these seemingly favorable behaviors had not resulted in satisfactory level of plaque control, as the mean PCR of the respondents was 49.5% at the initial examination. Among the respondents, however, those with more frequent toothbrushing (≥ 3× per day) and approximal cleaning habits were associated with better oral hygiene status. Any program or oral hygiene instruction intended to reduce caries and periodontal disease must focus on proximal and interdental areas [6]. It may be necessary to further emphasize interdental cleaning in the development of periodontal care plans.

We chose two conceptual models to study the determinants of oral self-care. These models were built on the knowledge base available so far and include aspects that have been neglected by use of singular health behavior model. The CSCCM implies that the dental hygienist works in dialogue with the patient in order to increase empowerment [15]. The dialogue results in a commitment where the patients set goals for themselves. The New Century model of oral health promotion [16] can be summarized as follows: oral health promotion is a function of oral health-related affect, behavior and cognition, time and situation. This model has been used as a framework for the analysis of oral self-care among adults with diabetes in Finland [24]. In our exploratory study, only 17% reported dental visits for a regular check-up within the last year. A similar result was reported in a previous study of a Japanese adult population [25]. Also in our study, 17% reported that they never followed or received professional advice on oral care. These behaviors fall short of the recommended levels. We found, however, that those who maintained regular check-ups and had closer perceived compliance with advice from dentists or dental hygienists had significantly better oral hygiene. These findings emphasize the importance of patient commitment in self-care. Our next step is to plan interventions based on the conceptual models, for the purpose of effectively assisting periodontal patients' self-care.

Attempts have been made to introduce behavioral cognitive approaches into patient care. Those efforts have shown some promising results in the oral health outcomes of patients with periodontitis [26,27]. A Chochrane systematic review identified tentative evidence from studies which demonstrated that psychological approaches to behavior management can improve oral hygiene related behaviors [20]. Recently, Syrjälä et al[28] reported the results of a comparative analysis in which psychological characteristics related to health behavior were examined for their ability to explain oral health habits, adherence to diabetes treatment, and measures of oral health. They found that self-efficacy was associated with oral health habits and diabetes adherence. The use of the present questionnaire allows clinicians to incorporate the essence of oral health models in a clinical setting. There are, however, limitations in all health behavior models used to explain the complexity of human health behavior [24]. There are also difficulties in developing effective strategies for improving long-term patient compliance with health promotion regimens, especially for chronic diseases and more so when lifestyle changes are necessary [6,26]. These points should be kept in mind when utilizing the conceptual models in treatment planning.

The application of oral health models to clinical situations has educational implications as well. Understanding the significance of preventing oral diseases and the basics of oral health promotion through biopsychosocial paradigm has become a primary concern of all dental and dental hygiene curricula [16,21,29]. Our clinic is part of the dental school hospital, and it is crucial to educate dental and dental hygiene students as well as young clinicians about the importance of patients' oral health behaviors and perceptions.

One weaknesses of this study would be the probable bias of the population, which is small in size and not necessarily represents the whole population with chronic periodontitis in Japan. We were unable to match or analyze the subject by gender in this pilot study. In a previous study in Japan, it has been reported that both age and gender differences can be a significant factor in oral self-care [30]. In the future study with larger sample size, we will evaluate the effect of age and gender differences. Validity and reliability of the questionnaire should be further examined. Additionally, the study only sought associations between patients self-care related behaviors and perceptions, and oral hygiene status at the pre-treatment stage. Another possible limitation is that patients may be hesitant or inconsistent in expressing their personal views about their health when self-reports are utilized [31].

We are in the process of analyzing the relationship between identified health care behaviors and periodontal parameters in larger patient poplulation. Also, our current effort is directed at elucidating the long-term relationship between patients' health beliefs, behaviors and oral health outcomes.

## Conclusion

In summary, the questionnaire facilitates the inclusion of multiple aspects of patient information, before initiation of periodontal treatment. There seems to be much room for improvement of oral hygiene and self-care among individuals presenting for an initial periodontal examination. Within the limits of the present study, the significant associations that were found between some of the self-care behaviors and oral hygiene levels document the important role of patient-centered oral health assessment in periodontal care.

## Abbreviations

CSCCM: Client Self-care commitment model; PD: probing depth; PCR: plaque control record

## Competing interests

The authors declare that they have no competing interests.

## Authors' contributions

AS designed the study, performed the data analyses, and drafted the manuscript. MK and FU contributed to data collection and analysis. SM, HH and HM collected data. TM oversaw procedures. All authors approved the manuscript.

## Pre-publication history

The pre-publication history for this paper can be accessed here:


